# Longitudinal Trajectories of Dietary Fibre Intake and Its Determinants in Early Childhood: Results from the Melbourne InFANT Program

**DOI:** 10.3390/nu15081932

**Published:** 2023-04-17

**Authors:** Fanney Thorsteinsdottir, Karen J. Campbell, Berit L. Heitmann, Miaobing Zheng

**Affiliations:** 1 Research Unit for Dietary Studies, The Parker Institute, Bispebjerg and Frederiksberg Hospital, 2000 Frederiksberg, Denmark; berit.lilienthal.heitmann@regionh.dk; 2 Institute for Physical Activity and Nutrition (IPAN), School of Exercise and Nutrition Sciences, Deakin University, Geelong, VIC 3220, Australia; karen.campbell@deakin.edu.au (K.J.C.); j.zheng@deakin.edu.au (M.Z.); 3 The Boden Group, Faculty of Medicine and Health, Sydney University, Sydney, NSW 2006, Australia; 4 The Department of Public Health, Section for General Medicine, University of Copenhagen, 1353 Copenhagen, Denmark

**Keywords:** fibre intake, trajectory, determinants, obesity

## Abstract

Background: Optimal nutrition during early childhood, including dietary fibre intake, is important for children’s health and development. Knowledge of fibre intake and its determinants in early childhood is limited. We aimed to describe fibre intake and sources and to identify trajectories of fibre intake at age 9, 18, 42, and 60 months and its child and maternal determinants. Associations between fibre trajectory groups and BMI z-scores and child overweight status were also assessed. Methods: This is a secondary analysis of longitudinal data from the Melbourne InFANT Program, trial registration: Current Controlled Trials (ISRCTN81847050). Group-based trajectory modelling was used to identify trajectories of fibre intake from ages 9 to 60 months (*n* = 503). Multivariable logistic or linear regression was used to assess the determinants of fibre intake trajectories and the association between fibre intake trajectories and obesity outcomes. Results: Four fibre intake trajectory groups were identified, with three groups following stable, rising trajectories of “Low” (52.3%), “Moderate” (32.2%), and “High” (13.3%), respectively. The remaining followed an “unstable” trajectory (2.2%). Girls versus boys were more likely to follow the “Low” fibre intake trajectory, whereas children who were breastfed for ≥6 months and whose mother had a university education were less likely to follow the “Low” fibre trajectory. No association was found between fibre trajectory groups and obesity outcomes. Conclusion: Most children followed a stable, rising trajectory of low fibre intake in early childhood. Child sex, breastfeeding duration and maternal education were significant determinants of low fibre intake trajectory.

## 1. Introduction

Optimal nutrition during early childhood is important for children’s growth, development, and future health [1]. Dietary fibres are carbohydrate polymers with three or more monomeric units (MU) that are neither digested nor absorbed in the human small intestine and include non-starch polysaccharides (NSP), resistant oligosaccharides (RO), resistant starch (RS), and lignin. The definition of dietary fibres differs between regions and expert groups; e.g., some only include NSP only and some define fibres as 10 or more MU. Adequate dietary fibre intake is important for healthy colonic function and for regulating blood cholesterol and blood glucose. Furthermore, a diet high in fibre-rich foods has been associated with decreased risk of chronic deceases such as type 2 diabetes, cardiovascular diseases, hypertension, cancer, and obesity among adults [2]. It has also been suggested that fibre intake modulates the gut barrier function [3], affecting gut immunity and inflammation. The associated health benefits of dietary fibre among children include lower risk of constipation [4,5], better cardiovascular health [6], lower obesity risk, fewer risk factors for diabetes [7], better cognitive control and memory [8], and creativity [9].

Dietary recommendations worldwide encourage the consumption of foods rich in fibre for all age groups. However, due to a lack of evidence, the fibre intake recommendation only provides Adequate Intake (AI). In Dietary Guidelines for Americans, AI for children aged 1–3 years is 19 g/day, and for children aged 2–3 and 4–8 years, the AI is 14 g/1000 kcal [10]. According to the European Food Safety Authority (EFSA), AI for children aged 1–3 and 4–6 years is 10 g/day and 14 g/day [11], respectively. In Australia, the recommended AI of dietary fibre for children 1–3 years old is 14 g/day and for 4–8 years old, it is 18 g/day [12]. No recommended AI has been set for children 0–12 months.

It is of concern that inadequate fibre consumption is common among children worldwide [13,14,15]. A US study showed that 3.2–9.1% of children aged 6–47.8 months met AI for fibre [16]. Furthermore, according to the US National Health and Nutation Examination Survey (NHANES), average fibre intake among children aged 2–5 years was 12 g/day in 2001–2002, and in the UK National Diet and Nutrition Survey (NDNS), the average fibre intake among children aged 1.5–3 years was 8 g/day [17]. Fayet-Moore et al. analysed the dietary fibre intake data from the Australian 2011–2012 National Nutrition and Physical Activity Survey [18,19] and found that 58.4% and 48.0% of children aged 2–3 years and 4–8 years, respectively, met the recommended AI of fibre, and that socio-economic status (SES) was a predictor of low fibre intake [18]. Furthermore, they reported that the main sources of fibre among children and adolescents aged 2–18 years were bread and bread rolls and mixed dishes such as sandwiches, burgers, pizza, tacos, pasta, and rice dishes [19]. The definition of dietary fibre might differ between the previously mentioned studies. To the best of our knowledge, no studies have reported fibre intake, sources of fibre, and predictors of fibre intake among children under two years nor examined changes in fibre intake (i.e., trajectories) in early childhood. Moreover, no studies investigated the association between changes in fibre intake and body mass index (BMI) z-score in early childhood.

Dietary habits acquired early in life tend to extend into later life [20]. Knowledge of fibre intake trajectories and their determinants in early childhood will be valuable for informing interventions to improve fibre intake. In a cohort of young Australian children, this study aimed to (1) describe fibre intake and sources over four time points for ages 9–60 months, (2) identify trajectories of fibre intake and their child and maternal determinants, and (3) assess the association between fibre trajectory groups and BMI z-score at age 60 months.

## 2. Materials and Methods

### 2.1. Study Population

This is a secondary analysis of longitudinal data from the Melbourne Infant Feeding Activity and Nutrition Trial (InFANT) Program [21]. The InFANT Program is a 15-month community-based cluster-randomized trial including first-time parents. The aim of the intervention was to reduce obesity risk behaviours of infants from ages 4 to 18 months. Children were followed up at ages 42 and 60 months. No additional intervention was provided after 18 months [22]. A total of 542 parent–child pairs from 62 first-time parent groups were included at baseline when infants were around four months of age. They participated in assessments at mid-intervention at age nine months, post-intervention at age 18 months, the first follow-up at age 42 months, and the second follow-up at age 60 months. The study design, intervention, and two follow-ups have been described in detail elsewhere [21,22,23]. Written informed consent was obtained from parents. Ethics approval was granted by the Deakin University Ethics Committee (ID number: EC 175-2007) and by the Victorian Office for Children (Ref: 80 CDF/07/1138).

### 2.2. Dietary Assessment

The child’s diet was assessed at ages 9, 18, 42, and 60 months using a telephone-administered 3-day 24 h recall with parents. Trained nutritionists contacted the parents on three non-consecutive days, including two weekdays and one weekend day. The parents were asked to recall all food and beverages the child had consumed in the previous 24 h; prior to the recall interview, the parents were provided with visual aids to assist with the estimation of portion size. In order to limit bias, the parents were contacted unscheduled (97%). One or two interviews with parents were scheduled for children that spent three days or more with another caregiver (e.g., daycare or grandparent). In case of a scheduled interview, the other caregiver, e.g., daycare or grandparent, was asked to record the child’s food and beverage intake using a purpose-designed food diary and deliver it to the parents; this was used by the parents during the recall interview. Trained researchers coded the data using the 2007 Australian Food and Nutrient Database (AUSNUT) Database [24] and an in-house developed database with additional products specific to infants to derive food and nutrient intake. A dietitian checked the coding of the data to ensure accuracy. The Multiple Source Method [25] was used to derive the usual intake from the three 24 h recalls. Fibre food sources were categorized into 33 groups (Supplementary Table S1) based on the standard food grouping in the Australian Food, Supplement and Nutrient Database (AUSNUT) from 2007 [24]. Food sources that provided at least 2% of the total mean fibre intake at one or more time points are presented in the current analysis.

### 2.3. Anthropometrics

A child’s birth weight was transcribed from the child’s health record. Trained staff measured length/height and weight at ages 9, 18, 42, and 60 months. All measurements were conducted using calibrated measurement tools. Length/height was measured to the nearest 0.1 cm, and weight was measured to the nearest 10 g. The children were wearing light clothing. Both length/height and weight were measured twice, and the average was registered. BMI z-scores were calculated using the World Health Organization’s sex-specific growth charts [26]. We categorized BMI z-scores to normal weight vs. overweight using the WHO overweight definition: overweight was defined as a BMI z-score > 2 for children <5 years and BMI z-score > 1 for children aged 5–19 years [27].

### 2.4. Child and Maternal Factors

Based on previous research and statistical significance, a number of child and maternal factors were assessed as covariates. Child sex (boy, girl), birth weight (low <2.5 kg, normal ≥2.5 kg), any breastfeeding duration (<6 months, ≥6 months), the timing of solid food introduction (<6 months, ≥6 months), maternal education (university (university degree and higher), pre-university (certificate/diploma/apprenticeship/high school)), maternal country of birth (Australia, another country), and maternal pre-pregnancy weight and height status (normal weight (<25 kg/m^2^), overweight or obese (≥25 kg/m^2^)). Information on child sex and maternal factors was collected at baseline using a parent self-administered questionnaire. Information on breastfeeding duration and timing of solid food introduction was collected at baseline (age four months) and follow-up (age 9 and 18 months). Breastfeeding duration was obtained by combining responses to two breastfeeding-related questions, “How long did you breastfeed your child?” giving the response options “never breastfed”, “still breastfeeding,” and “stopped breastfeeding”, and an open question “Age of the baby in months and in weeks when you stopped breastfeeding” administered at ages 3, 9 and 18 months. Timing of solid food introduction was obtained based on a question at baseline, “Have you started to give your baby solids (food other than milk)?” giving the options “yes” or “no”, and an open question at baseline and 9 months follow-up “How old was your baby in months and in weeks when you started giving them solids?”. Consistent with the previous analysis, breastfeeding duration and timing of solid food introduction were analysed as categorical variables using the cut-off of six months [28].

### 2.5. Statistical Analysis

Descriptive statistics are presented as number (n) and percentages (%) for categorical variables and n, mean and standard deviation (SD) for continuous variables. We provide descriptive statistics for the baseline covariates, total fibre intake, and main fibre food sources at each time point. A comparison of fibre intake and obesity outcomes between intervention and control groups revealed no statistical difference. Therefore, data from the intervention and control groups were pooled for the present analysis, and intervention allocation was included as a covariate.

Group-based trajectory modelling was used to identify trajectory groups of fibre intake from ages 9 to 60 months using the “Traj” command in Stata. Individuals with fibre intake measured at one or more time points over the four time points were included. Analyses were also conducted for children with two or more fibre intake measurements (n = 420) and showed similar results (Supplementary Table S2). Thus, analysis with one or more fibre measurements was presented as the primary analysis. Censored normal models with fibre intake as the dependent variable and linear, quadratic, and cubic function of age in months as the independent variable were conducted. We fitted and compared models with up to five trajectory groups. The identification of the optimal number of trajectory groups is contingent on model fit Bayesian Information Criterion (BIC), average posterior probability >0.7), model parsimony, visual inspection of the distinctiveness of trajectory groups, and clinical interpretability. Higher (less negative) BIC indicates a better model fit [29]. Fibre intake trajectory groups were then used in the subsequent analysis as a categorical outcome variable for assessing child and maternal predictors.

Multivariable logistic regression models were performed to examine the association between child and maternal predictors of fibre trajectory groups. Given the high prevalence of low fibre intake in children and the adverse effect of low fibre intake on health, determinants of low fibre intake trajectory were assessed. Other trajectory groups were combined and analysed as the low fibre intake trajectory reference category. The following determinants were assessed: child sex, birth weight, breastfeeding duration, the timing of solid food introduction, maternal employment status, education, country of birth, and pre-pregnancy body weight status. Multi-collinearity between these factors was examined, and no significant correlation was found.

We conducted a multivariable linear and logistic regression model to examine the association of fibre trajectory groups with child BMI z-score and overweight status at age 60 months, respectively. In model 1, we adjusted for BMI z-score at age nine months; in model 2, we additionally adjusted for the treatment group, child sex, breastfeeding duration, timing of solid food introduction, maternal employment status, education, country of birth and pre-pregnancy BMI; and model 3 additionally adjusted for energy intake at age nine months. As children transition from a milk-based diet to a food-based diet by age one year, sensitivity analyses excluding the 9 months old fibre intake data were conducted.

All statistical analyses were performed using Stata version 15 (StataCorp. 2017. Stata Statistical Software: Release 15. College Station, TX, USA, www.stata.com (accessed on 14 April 2023)). Codes are available upon request. The statistical tests were two-sided at a 5% significance level.

## 3. Results

### 3.1. Characteristics of the Study Population

**Figure 1:**
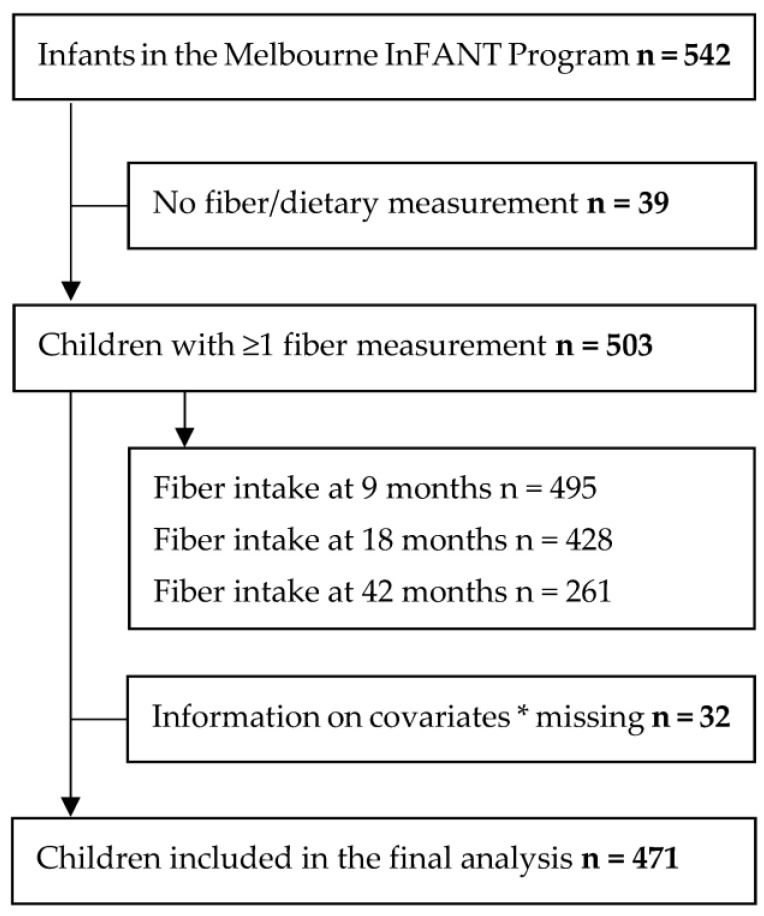
Flowchart of the study population. * child sex, birthweight, breastfeeding, introduction to solid food, and maternal employment status, education, country of birth and weight status, intervention group but excluding the variable assessed.

Of the 542 children that participated at baseline, 503 children with one or more fibre intake measurements over four time points were included in the current analysis (Figure 1). The cohort characteristics are presented in Table 1. The mean child age at a mid-intervention, postintervention, first follow-up, and second follow-up was 9.2, 18.0, 43.3, and 60.7 months respectively. The majority of the mothers were unemployed, born in Australia, and about two-thirds of the mothers were overweight or obese prior to pregnancy. Most of the children were breastfed for more than six months but were introduced to solid food before the age of six months. Baseline characteristics did not differ between those included in the analysis (n = 503) and those excluded from the analysis (n = 39).

### 3.2. Fibre Intake

The mean fibre intake was 8.7, 13.0, 16.1, and 18.3 g/day at ages 9, 18, 42, and 60 months, respectively (Table 2). At age 42 months, 60.5% of the children had fibre intake greater than AI according to Nutrient Reference Values for Australia and New Zealand, whereas at ages 18 and 60 months, only 35.8% and 40.4%, respectively, had intake greater than AI. Fibre density increased slightly with age ranging from 10.1 g/1000 kcal at age 9 months to 13.2 g/1000 kcal at age 60 months. The main sources of fibre were fruits and whole-grain bread and/or cereals in all age groups except at age nine months, where fruits and vegetables provided the main sources of fibre (Table 3). The proportion of fibre from energy-dense, low-fibre foods, including refined bread and/or cereals and cakes and/or cookies, increased with age.

### 3.3. Fibre Intake Trajectory

**Figure 2:**
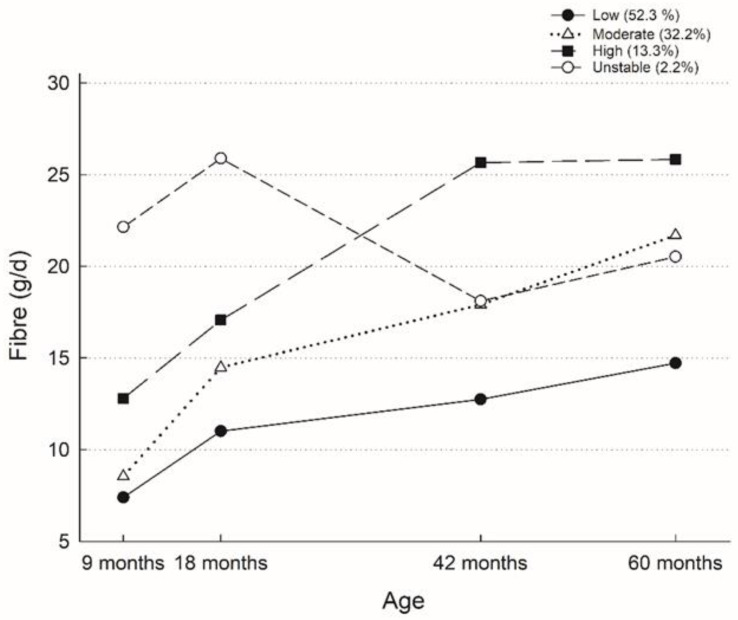
Fibre trajectory groups in the Melbourne Infant Feeding Activity and Nutrition Trial Program, including those with one or more fibre measurements (n = 503).

BIC, as noted above, relates to model fit where higher values indicate better model fit, which improved from the two-group to the four-group model (Supplementary Table S3). The five-group and four-group models revealed a very similar BIC. The five-, four-, and three-group models revealed a consistent “unstable” fibre trajectory that consisted of 2.2% of the total sample only. For model parsimony, the four-group model was retained. Three out of four groups revealed an increase in fibre intake from age 9 to 60 months and were labelled as “Low” (52.3%), “Moderate” (32.2%), and “High” (13.3%) (Figure 2). The remaining 2.2% showed an unstable fibre trajectory, with fibre intakes consistently higher than the “Low” fibre intake trajectory group. The “Moderate”, “High”, and “Unstable” fibre intake trajectory groups were combined for subsequent logistic regression to evaluate the predictors of the “Low” fibre intake trajectory group.

### 3.4. Child and Maternal Predictors of Low Fibre Trajectory Groups

Results from the multivariable logistic regression analysis showed that low fibre intake trajectories were more likely among girls (OR 1.67, 95%CI 1.13, 2.46); and less likely in children who were breastfed for six months or more (OR 0.44, 95%CI 0.30, 0.72) and in children whose mother had a university education (OR 0.66, 95%CI 0.44, 0.98). Birth weight, the timing of solid food introduction, maternal working status, pre-pregnancy BMI status, and country of birth were not associated with the risk of having a low fibre intake trajectory nor did the intervention (Table 4). Sensitivity analysis excluding fibre intake at age 9 months gave essentially the same results.

### 3.5. Fibre Trajectory Groups and BMI z-Score at Age 60 Months

We found no association between fibre trajectory groups and overweight status or BMI z-score at age 60 months. The predicted mean BMI z-score in the low fibre trajectory group was 0.50 (95%CI 0.38–0.61) and 0.62 (95%CI 0.48–0.76) in the moderate to high fibre trajectory group in the fully adjusted model (Supplementary Table S4), and 22.3% of the children were overweight. The moderate to high fibre trajectory group had a slightly higher predicted mean BMI z-score; however, this was not statistically significant (Table 5). We saw the same trend, also not statistically significant, for overweight status. Sensitivity analysis excluding fibre intake at age 9 months gave essentially the same results.

## 4. Discussion

In a cohort of Australian children, we describe fibre intake and sources in early childhood. Our study is the first to identify fibre intake trajectories and their determinants in early childhood. Most children followed stable, rising trajectories of “Low”, “Moderate”, and “High” fibre intake from ages 9 to 60 months. Child sex, breastfeeding duration, and maternal education were the significant determinant of the “Low” fibre intake trajectory. However, no association was found between fibre intake trajectory groups and obesity outcomes.

The fibre intake in this cohort was similar to those reported in other Australian studies [18,30] and studies from other countries [17]. On average, less than half of the children aged 18 to 60 months met the recommended AI for fibre intake. Currently, there is no recommended AI for 9 months old, as infants are expected to have a predominantly milk-based diet and there is a lack of dietary intake data during infancy. Consistent with findings from other studies [17,19], the main sources of fibre were fruits, vegetables, and cereal products. In our study, the main source of fibre changed from fruits and vegetables at age 9 months to fruits and whole-grain bread and/or cereals from age 18 to 60 months. We also observed that the proportion of fibre from refined bread and/or cereals and cakes and/or cookies increased with age, indicating that consuming foods high in micronutrients and fibres are replaced by energy-dense foods that are low in micronutrients. Fruits and vegetables are the main sources of soluble fibre, whereas the main source of insoluble fibre is cereals and whole-grain products [17]. Intake of insoluble non-fermentable fibres has been associated with a reduced risk of developing type 2 diabetes among adults [31]. Among children, fibre intake from fruit and vegetables has been associated with a lower cardiometabolic risk score [6]. Soluble fibres are highly fermentable in the colon, where they are fermented by the gut microbiota, and dietary fibre intake has been shown to beneficially influence the composition and response of the gut microbiota [32,33]. Given the differential impact of different types of fibre, such as soluble and insoluble fibre, on health outcomes, it would be desirable to study them separately. However, most food items rich in fibre contain a mixture of fibre types [17], making it difficult to assess them separately. More importantly, data on sub-division according to the different fibre types is not readily available [17]. Therefore, in order to explore the link between specific fibre types and health outcomes, we need a more detailed classification of fibre in food.

It is expected to observe an increase in fibre intake from ages 9 to 60 months given children transitions from a milk-based diet to a food-based diet. Our finding that children followed stable, rising trajectories of fibre intake in early childhood aligns with studies that revealed tracking of food preferences across life stages [20,34]. This indicates that changing dietary habits established at 9 months is difficult, making it important to start dietary interventions very early in life. The determinants of low fibre intake in our study were the child’s sex (girl), mothers not having a university education, and breastfeeding duration of less than six months. Similarly, Fayet-Moore et al. found that Australian children aged 2–18 years of high socioeconomic status (SES) were more likely to meet the recommended AI for fibre intake, and a higher proportion of boys than girls met the recommendations [18]. Higher fibre intake among children of mothers with a university education could be explained by higher nutritional knowledge and a better home food environment among mothers with higher education [35]. The intake of fibre-dense foods might partly explain the difference between boys and girls. In our cohort, boys had higher intakes of fibre-dense foods (fruits, whole-grain bread, and cereal) than girls. Previous studies have suggested that children usually prefer energy-dense, sugary and salty food (e.g., refined bread and cereals, cakes, and cookies that are low in fibre) over foods that are sour and bitter (e.g., many fruits and vegetables that are high in fibre) and that children that are breastfed are more willing to try new foods and are less picky as they are exposed to different flavours through breastmilk [20,34]. This could explain why in our study, we see that children that are breastfed for less than six months were more likely to be low-fibre consumers.

Fibre intake may be beneficial for weight control due to its effect on satiety and the positive effect on microbiota [36]. Previous studies among children have been inconsistent, showing either no evidence that fibre from grain reduces the risk of being overweight or that fibre density increases the risk of being overweight [7]. Our study did not find any association between fibre intake trajectory and BMI z-score at age 60 months. It is plausible that low dietary fibre has more long-term effects and that more long-term follow-up is needed; however, it is also plausible that dietary patterns such as consumption of energy-dense, high-fat and low-fibre foods are more important for weight control than only a low intake of fibre.

There are several strengths to our study. Our study is the first to report on fibre intake trajectories and predictors of fibre intake in young Australian children. We assessed diet with a 3-day, 24 h recall, which provides high-quality dietary intake data, although reporting bias cannot be dismissed. The longitudinal study design with repeated dietary measurements permitted the use of group-based trajectory modelling to identify heterogeneous fibre intake trajectory groups within the study sample without excluding people with missing data. Our study is not without weaknesses. Most mothers in our study had a university education, which limits the generalizability of our results to the general population of Australian children. We were not able to distinguish between different types of fibre (e.g., soluble vs. insoluble), which is of interest due to their different physicochemical properties. This will require food composition databases that distinguish between different types of fibre. It would have been desirable to adjust for maternal diet and fibre intake in our multivariable models; however, we did not have information on maternal diet.

Adequate fibre intake among children is important for children’s development and for current and future health. Diet is a modifiable risk factor for adverse health outcomes. Our results suggest that fibre intake tracks over time; children with low, moderate, and high fibre intake at 9 months remained in their respective fibre intake group at 60 months. Results from our study have important implications for policy and practice. As a result of limited data in early childhood, current fibre recommendations provide AI only, which was based on the median observed intake in a healthy population [11]. Our study findings will contribute evidence to future refinement of fibre intake recommendations. A general high prevalence of low fibre intake underscores the need to promote adequate fibre intake early in life. Results from our study and previous studies suggest that interventions should focus on children early in life or as early as during pregnancy, as taste preferences are established through the amniotic fluid, breastfeeding, and weaning [20,34]. Furthermore, it might be beneficial to introduce more fibre-rich food sources into children’s diets during infancy to set up their taste preferences. Findings on determinates of fibre intake trajectories provide valuable information on at-risk groups and modifiable risk factors for future intervention and strategies to increase fibre intake.

## 5. Conclusions

The majority of children aged 9 to 60 months did not meet the recommended AI for dietary fibre intake, and sources of fibre intake changed over time from fruit and vegetables to fruits and whole-grain bread and/or cereals. Four distinctive fibre intake trajectory groups were identified, with most children following stable, rising fibre intake trajectories of low, moderate, or high. About half of the sample followed the low fibre intake trajectory. In order to promote the child’s future health, promoting a fibre-rich diet, especially among parents of low SES, is important early in life and preferably before age nine months.

## Figures and Tables

**Table 1:** Characteristics in the Melbourne Infant Feeding Activity and Nutrition Trial Program (n = 503).

	n	% or Mean (SD)
Intervention group		
Intervention	257	51.1
Control	246	48.9
Child sex		
Girl	235	46.7
Boy	268	53.3
Child age in months at each time point		
9 months	503	9.2 (1.1)
18 months	486	17.9 (1.7)
42 months	359	43.3 (2.5)
60 months	360	60.7 (1.6)
Child BMI z-score		
9 months	502	0.15 (0.98)
18 months	466	0.82 (0.99)
42 months	353	0.63 (0.87)
60 months	361	0.53 (0.97)
Birthweight		
Low (<2.5 kg)	35	7.0
Normal (≥2.5 kg)	462	91.8
Missing	6	1.2
Any breastfeeding duration		
<6 months	175	34.8
≥6 months	317	63.0
Missing	11	2.2
Timing of solids food introduction		
Before <6 months	337	67.0
At and after ≥6 months	141	28.0
Missing	25	5.0
Maternal employment status		
Employed	45	8.9
Unemployed	450	89.5
Missing	8	1.6
Maternal education		
University	272	54.1
Preuniversity	228	45.3
Missing	3	0.6
Maternal weight status		
Normal weight	181	36.0
Overweight or obese	318	63.2
Missing	4	0.8
Maternal country of birth		
Australia	396	78.7
Other	104	20.7
Missing	3	0.6

**Table 2:** Fibre intakes (grams per day), fibre density (grams per MJ), energy intake (MJ per day) at ages 9, 18, 42, and 60 months) in the Melbourne Infant Feeding Activity and Nutrition Trial Program.

	9 Months (n = 495)	18 Months (n = 428)	42 Months (n = 261)	60 Months (n = 270)
	Mean ± SD			
Fibre intake (g/day)	8.7 ± 4.5	13.0 ± 4.4	16.1 ± 5.3	18.3 ± 5.3
Fibre density (g/MJ)	2.4 ± 1.0	2.9 ± 0.8	3.0 ± 0.8	3.1 ± 0.8
Energy intake (MJ/day)	3.6 ± 0.9	4.5 ± 0.9	5.4 ± 1.2	5.9 ± 1.2
	Median (IQR)			
Fibre intake (g/day)	7.7 (5.5–10.8)	12.5 (9.7–15.6)	14.9 (12.6–18.7)	17.6 (14.5–21.6)

**Table 3:** Main fibre food sources at ages 9, 18, 42, and 60 months in the Melbourne Infant Feeding Activity and Nutrition Trial Program.

		9 Months (n = 495)			18 Months (n = 428)			42 Months (n = 261)			**60 Months (n = 270)**	
Food Group ^a^	% ^b^	% Total Fibre	Total Fibre (g)	% ^b^	% Total Fibre	Total Fibre (g)	% ^b^	% Total Fibre	Total Fibre (g)	% ^b^	% Total Fibre	Total Fibre (g)
		Mean ± SD			Mean ± SD			Mean ± SD			Mean ± SD	
Vegetables	92	24.2 ± 17.2	2.1 ± 2.1	89	12.6 ± 11.0	1.6 ± 1.7	92	10.6 ± 7.6	1.7 ± 1.4	94	12.7 ± 9.1	2.4 ± 1.9
Fruit	95	23.5 ± 15.7	2.1 ± 1.8	99	27.1 ± 12.6	3.6 ± 2.0	99	26.2 ± 12.1	4.3 ± 2.5	99	24.8 ± 11.1	4.7 ± 2.7
Breads/cereals—whole-grain	83	16.5 ± 14.6	1.4 ± 1.4	97	26.9 ± 13.4	3.6 ± 2.2	94	26.9 ± 14.4	4.4 ± 2.9	96	25.1 ± 14.7	4.7 ± 3.3
Breads/cereals—refined	74	4.8 ± 7.1	0.4 ± 0.8	94	8.8 ± 8.5	1.0 ± 1.0	98	10.7 ± 9.1	1.6 ± 1.4	96	11.8 ± 9.4	2.0 ± 1.5
Infant foods	61	9.5 ± 13.9	0.7 ± 1.1	36	2.2 ± 5.3	0.3 ± 0.6	8	0.7 ± 3.5	0.1 ± 0.5	3	0.2 ± 1.7	0.0 ± 0.3
Infant cereals	73	6.7 ± 10.1	0.5 ± 0.8	20	0.6 ± 2.4	0.1 ± 0.2	1	0.0 ± 0.5	0.0 ± 0.1	0	-	-
Red meat mixed dishes	34	2.7 ± 6.8	0.2 ± 0.7	48	2.2 ± 4.5	0.3 ± 0.6	39	1.3 ± 2.5	0.2 ± 0.4	37	1.3 ± 2.7	0.2 ± 0.5
Potatoes	39	2.0 ± 4.2	0.2 ± 0.4	49	2.6 ± 4.7	0.3 ± 0.5	66	3.8 ± 5.3	0.6 ± 0.7	66	3.7 ± 4.9	0.6 ± 0.8
Cakes/Cookies	25	0.6 ± 1.8	0.0 ± 0.2	72	2.6 ± 3.3	0.3 ± 0.4	81	3.8 ± 4.2	0.6 ± 0.6	85	3.8 ± 4.2	0.7 ± 0.7
Legumes	17	2.1 ± 6.0	0.2 ± 0.7	25	2.8 ± 6.8	0.5 ± 1.2	23	2.1 ± 5.4	0.4 ± 1.2	21	2.3 ± 6.5	0.5 ± 1.5
Pasta—refined	41	2.0 ± 4.0	0.2 ± 0.4	55	3.0 ± 4.7	0.4 ± 0.6	54	3.2 ± 4.9	0.5 ± 0.7	49	2.9 ± 5.0	0.5 ± 0.8

^a^ food sources that provided at least 2% of total fibre at one or more time points are presented, ^b^ percentage of children who consumed food items in the food group.

**Table 4:** Determinants of low dietary fibre trajectory in the Melbourne Infant Feeding Activity and Nutrition Trial Program (n = 471).

	Univariable	Multivariable ^a^
	OR (95% CI)	
Child sex (girl vs. boy)	1.56 (1.08, 2.27)	1.67 (1.13, 2.46)
Birthweight (<2.5 vs. ≥2.5 kg)	1.53 (0.71, 3.29)	1.13 (0.51, 2.51)
Breastfeeding duration (≥ 6 vs. <6 months)	0.45 (0.30, 0.68)	0.47 (0.30, 0.72)
Introduction to solid food (before 6 vs. after 6 months)	0.73 (0.48, 1.10)	0.69 (0.45, 1.07)
Maternal employment status (yes vs. no)	1.49 (0.75, 2.95)	1.45 (0.72, 2.94)
Maternal education (university vs. non-university)	0.59 (0.40, 0.86)	0.66 (0.44, 0.98)
Maternal pre-pregnancy BMI (≥25 kg/m^2^ vs. <25 kg/m^2^)	1.43 (0.97, 2.11)	1.25 (0.83, 1.90)
Mother not born in Australia vs. born in Australia	1.15 (0.72, 1.81)	1.29 (0.79, 2.09)
Intervention	0.91 (0.63, 1.31)	0.84 (0.57, 1.23)

^a^ multivariable model included the variables child sex, birthweight, breastfeeding, introduction to solid food, and maternal employment status, education, country of birth and weight status, intervention group but excluding the variable assessed.

**Table 5:** Determinants of low dietary fibre trajectory in the Melbourne Infant Feeding Activity and Nutrition Trial Program (n = 471).

BMI z-Score *	Mean Δ (95%CI)	*p*
Model 1 (n = 345)	−0.10 (−0.28–0.08)	0.27
Model 2 (n = 345)	−0.12 (−0.30–0.07)	0.20
Model 3 (n = 342)	−0.12 (−0.31–0.07)	0.20
Child Overweight Status *	**OR (95%CI)**	***p***
Model 1 (n = 345)	1.00 (0.57–1.78)	0.96
Model 2 (n = 345)	0.94 (0.51–1.73)	0.85
Model 3 (n = 342)	0.94 (0.51–1.73)	0.84

* low fibre intake trajectory group compared to high and unstable fibre intake trajectory group, Model 1: adjusted for BMI z-score at 9 months, Model 2: additionally adjusted for the treatment group, child sex, breastfeeding duration, the timing of solid food introduction, maternal employment status, education, country of birth and pre-pregnancy BMI upon model 1, Model 3: additionally adjusted for total energy intake upon model 2.

## Data Availability

Data used for current analyses are not publicly available because of ethical considerations due to potential identifying information. Data are available from request with a methodologically sound proposal via gavin.abbott@deakin.edu.au and pending approval from the relevant ethics committees.

## Supplementary Materials

The following supporting information can be downloaded at: https://www.mdpi.com/article/10.3390/nu15081932/s1, Table S1: Categories of fibre food sources; Table S2: Determinants of low dietary fibre trajectory in the Melbourne Infant Feeding Activity and Nutrition Trial Program, including only those with fibre measurement at two or more time points (n = 420), Table S3: Model fit, Table S4: Predicted mean BMI z-score and mean difference in BMI z-score at age 60 months among children in the low fiber intake trajectory group compared to high and unstable fiber intake trajectory group.
