# Supplementary material for: Longitudinal Trajectories of Dietary Fibre Intake and Its Determinants in Early Childhood: Results from the Melbourne InFANT Program
Source: Nutrients. 2023 Apr 17;15(8):1932. doi: 10.3390/nu15081932 (PMC10145308; doi:10.3390/nu15081932)
Supplement: Supplementary file 1 [file nutrients-15-01932-s001.zip › Thorsteinsdottir_Supplementary_Table S1.pdf]

Supplementary Table S1. Categories of fibre food sources

**1.**	fibre from accompaniments
**2.**	beef/veal/lamb
**3.**	refined breads & cereals
**4.**	wholegrain breads & cereals
**5.**	breast milk
**6.**	butter/oil/fat spreads
**7.**	cakes/biscuits
**8.**	dietary product
**9.**	egg & egg dishes
**10.**	fish
**11.**	fruit
**12.**	infant cereals/products
**13.**	infant drinks
**14.**	infant foods
**15.**	infant/toddler formula
**16.**	legumes
**17.**	milk & milk products
**18.**	non-milk drinks
**19.**	nuts & seeds
**20.**	organ meat
**21.**	refined pasta
**22.**	wholegrain pasta
**23.**	potatoes
**24.**	poultry
**25.**	processed meat
**26.**	savoury snacks
**27.**	soup
**28.**	soy yoghurt/milk substitute
**29.**	sugar-based accompaniments
**30.**	fruit/vegetable juice/drink
**31.**	sugar-sweetened beverages
**32.**	sweet snacks
**33.**	vegetables
