# Supplementary material for: Longitudinal Trajectories of Dietary Fibre Intake and Its Determinants in Early Childhood: Results from the Melbourne InFANT Program
Source: Nutrients. 2023 Apr 17;15(8):1932. doi: 10.3390/nu15081932 (PMC10145308; doi:10.3390/nu15081932)
Supplement: Supplementary file 1 [file nutrients-15-01932-s001.zip › Thorsteinsdottir_Supplementary_Table S2.pdf]

Supplementary Table S2. Determinants of low dietary fibre trajectory in the Melbourne Infant Feeding Activity and Nutrition Trial Program, including only those with fibre measurement at two or more time points (***n***=420)

	Unadjusted	Adjusted[a]
	OR (95% CI)	
Child sex (girl vs body)	1.6 (1.1, 2.3)	1.6 (1.1, 2.5)
Birthweight (<2.5 vs ≥2.5kg)	1.3 (0.6, 2.9)	1.1 (0.5, 2.5)
Breastfeeding duration (≥ 6 vs <6 months)	0.4 (0.3, 0.6)	0.5 (0.3, 0.7)
Introduction to solid food (before 6 vs after 6 months)	0.8 (0.5, 1.2)	0.7 (0.5, 1.2)
Maternal employment status (yes vs no)	1.5 (0.8, 3.0)	1.8 (0.8, 3.8)
Maternal education (university vs non-university)	0.6 (0.4, 0.9)	0.7 (0.5, 1.1)
Maternal pre-pregnancy BMI (≥25kg/m2 vs <25kg/m2)	1.4 (0.9, 2.1)	1.2 (0.8, 1.9)
Mother not born in Australia vs born in Australia	1.3 (0.8, 2.0)	1.4 (0.9, 2.4)
**Intervention**	0.9 (0.6, 1.3)	0.9 (0.6, 1.3)

[a]adjusted model included all variables (child sex, birthweight, breastfeeding, introduction to solid food, and maternal employment status, education, country of birth and weight status) excluding the variable assessed, and intervention group.
