# Supplementary material for: Longitudinal Trajectories of Dietary Fibre Intake and Its Determinants in Early Childhood: Results from the Melbourne InFANT Program
Source: Nutrients. 2023 Apr 17;15(8):1932. doi: 10.3390/nu15081932 (PMC10145308; doi:10.3390/nu15081932)
Supplement: Supplementary file 1 [file nutrients-15-01932-s001.zip › Thorsteinsdottir_Supplementary_Table S3.pdf]

Supplementary Table S3. Model fit

Trajectory groups	BIC[a]	AIC[b]	Estimated probabilities (%)[d]				
			1	2	3	4	5
**1**	-4346	-4336	100				
**2**	-4266	-4246	75	25			
**3**	-4245	-4213	27	71	2		
**4**	-4243	-4201	52	32	14	2	
**5**	-4241	-4188	49	34	1	14	2

[a]Bayesian Infromation Criterion, [b]Akaike Information Criteria, [d]estimated % in each group
