# Supplementary material for: Longitudinal Trajectories of Dietary Fibre Intake and Its Determinants in Early Childhood: Results from the Melbourne InFANT Program
Source: Nutrients. 2023 Apr 17;15(8):1932. doi: 10.3390/nu15081932 (PMC10145308; doi:10.3390/nu15081932)
Supplement: Supplementary file 1 [file nutrients-15-01932-s001.zip › Thorsteinsdottir_Supplementary_Table S4.pdf]

Supplementary Table S4. Predicted mean BMI z-score and mean difference in BMI z-score at age 60 months among children in the low fiber intake trajectory group compared to high and unstable fiber intake trajectory group

	**High fiber trajectory BMI z-score (95%CI)**	**Low fiber trajectory BMI z-score (95%CI)**	**Mean Δ (95%CI)**	**p**
Model 1 (n=345)	0.60 (0.47-0.74)	0.50 (0.39-0.62)	-0.10 (-0.28-0.08)	0.27
Model 2 (n=345)	0.61 (0.48-0.75)	0.49 (0.38-0.61)	-0.12 (-0.30-0.07)	0.20
Model 3 (n=342)	0.62 (0.48-0.76)	0.50 (0.38-0.61)	-0.12 (-0.31-0.07)	0.20

Model 1: adjusted for BMI z-score at 9 months, Model 2: additionally adjusted for treatment group, child sex, breastfeeding duration, timing of solid food introduction, maternal employment status, education, country of birth, and pre-pregnancy BMI upon model 1, Model 3: additionally adjusted for total energy intake upon model 2
